# A hybrid inorganic–organic light-emitting diode using Ti-doped ZrO_2_ as an electron-injection layer†

**DOI:** 10.1039/c8ra00259b

**Published:** 2018-02-22

**Authors:** Jayaraman Jayabharathi, Sekar Panimozhi, Venugopal Thanikachalam, Annadurai Prabhakaran, Palanivel Jeeva

**Affiliations:** Department of Chemistry, Annamalai University Annamalainagar 608 002 Tamilnadu India jtchalam2005@yahoo.com +91 9443940735

## Abstract

We have fabricated stable efficient iridium(iii)-bis-5-(1-(naphthalene-1-yl)-1*H*-phenanthro[9,10-*d*]imidazole-2-yl) benzene-1,2,3-triol (acetylacetonate) [Ir(NPIBT)_2_ (acac)] doped inverted bottom-emissive green organic light-emitting diodes using Ti-doped ZrO_2_ nanomaterials as the electron injection layer. The current density (*J*) and luminance (*L*) of the fabricated devices with Ti-doped ZrO_2_ deposited between an indium tin oxide cathode and an Ir(NPIBT)_2_ (acac) emissive layer increased significantly at a low driving voltage (V) compared with control devices without Ti-doped ZrO_2_. The Ti-doped ZrO_2_ layer can facilitate the electron injection effectively and enhances the current efficiency (*η*_c_) of 2.84 cd A^−1^ and power efficiency (*η*_p_) of 1.32 lm W^−1^

## Abstract 2

Ti-doped ZrO_2_ facilitates electron injection effectively, leading to enhanced current efficiency of 2.84 cd A^−1^ and power efficiency of 1.32 lm W^−1^

## Graphical abstract

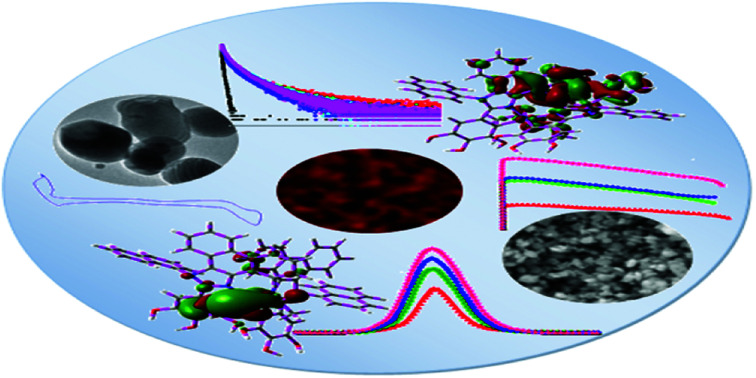

## 1. Introduction

Great research effort has been directed to the area of organic light emitting diodes (OLEDs) to enhance device stability and efficiency for future display applications.^1–4^ However, environmental stability and efficiency are the major issues for the development of device structures.^5–8^ The low work function metals, barium or calcium as electron injecting layers (EILs) may easily degrade in the presence of oxygen and moisture.^9,10^ Due to low-cost, visible-light transparency, environmental stability, carrier transport properties and tuning film morphology to micrometer scales of metal oxides they are both attractive candidates in OLEDs.^11–15^ They are used as hole (HIL)/electron (EIL) injection layers in hybrid organic–inorganic light-emitting diodes (HyLEDs).^11,12^ Titanium dioxide^11,12,16^/zinc oxide^13,17–19^ films are employed as EIL where as molybdenum trioxide^11,13^ is used as HIL in HyLEDs. HyLEDs based on poly(9,9-dioctylfluorene-altenzothiadiazole) (F8BT) as an electroluminescent layer combined with ZnO and MoO_3_ as EIL and HIL, respectively, exhibit maximum luminance (6500 cd m^−2^).^13^ Efficiencies of HyLEDs were tuned by both hole/electron injection from metal oxide EIL into *E*_LUMO_ of emissive layer.^20–28^ Bolink *et al.*, fabricated ITO/TiO_2_/F8BT/MoO_3_/Au HyLEDs results poor efficiences due to (i) higher energy barrier for injection from ITO to TiO_2_ and (ii) poor hole blocking functionality of titania.^29^ Therefore, it is urgent need to find alternate EILs which enable effective electron injection into emissive layer *E*_LUMO_.^30^ Herein, we present HyLEDs based on newly synthesized electron injection layer Ti-doped ZrO_2_ and Ir(NPIBT)_2_ (acac) as emitting layer. The efficiencies of HyLEDs imply that Ti-doped ZrO_2_ is an potential carrier injection material with reduced wt% improves the efficiencies. The improved HyLEDs performances compared to previous findings show that our device structure can be used to harvest efficient electroluminescence.

## 2. Materials and instrumental techniques

The structure of emissive materials was confirmed with ^1^H/^13^C NMR and mass spectra, recorded with Bruker 400 MHz spectrometer and Agilent (LCMS VL SD), respectively. Oxidation potentials were measured from potentiostat CHI 630A electrochemical analyzer. The Lambda 35 and Lambda 35 spectrophotometer with integrated sphere (RSA-PE-20) instrument (PerkinElmer) was employed to measure absorbance in both solution and film states. Emissive properties (PL) were analyzed with PerkinElmer fluorescence spectrometer (LS55) measurement. Thermal characteristics such as decomposition (*T*_d_) and glass transition (*T*_g_) temperatures was analyzed with PerkinElmer thermal analysis system (10 °C min^−1^; N_2_ flow rate of 100 mL min^−1^) and NETZSCH-DSC-204 (10 °C min^−1^ under N_2_ atmosphere), respectively. The PL QY (quantum yield) was measured using quinine sulphate (0.54) as reference [
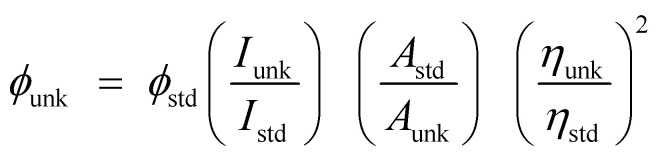
: *ϕ*_unk_ is unknown material QY, *ϕ*_std_ is standard QY, *I*_unk_ is unknown material emission intensity, *I*_std_ is standard emission intensity, *A*_unk_ is unknown sample absorbance, *A*_std_ is standard absorbance, *η*_unk_ is unknown material refractive index and *η*_std_ is standard refractive index]. The TCSPC (time correlated single photon counting) results fit to *f*(*t*) = *α* exp (−*t*/*τ*), [mono exponential decay: *α* – pre-exponential factor and *τ* lifetime of various excited states]. The chemical composition of Ti-doped ZrO_2_ was determined with X-ray photoelectron spectra: ESCA^−3^ Mark II spectrometer-VG – Al Kα (1486.6 eV) radiation. JEOL JSM-5610 equipped with back electron (BE) detector and FEI Quanta FEG was used to record energy dispersive X-ray spectra (EDS). Philips TEM with 200 kV electron beam was used to record TEM (transmission electron microscopy) image of the nanomaterials and SAED (selected area electron diffraction) pattern was taken from Philips TEM with CCD camera (200 kV). Equinox 1000 diffractometer using Cu Kα rays (1.5406 Å; current – 30 mA; 40 kV) was employed to record powder XRD.

### 2.1. Fabrication of OLED

The fabricated green OLEDs with a configuration: ITO/Ti–ZrO_2_/Ir(NPIBT)_2_ (acac)/MoO_3_/Au, where ITO and Au are used as cathode and anode conductors, respectively. Ir(NPIBT)_2_ (acac) and MoO_3_ are used as emissive layer and barrier-reducing HIL and electron-blocking layer, respectively. All the layers were deposited on ITO plate by thermal evaporation unit with glove box under optimized evaporation rates. The thicknesses have been monitored using quartz crystal digital thickness monitor. The current density–voltage and light intensity of HyLEDs were measured using Keithley 2400 source measuring unit. The EL spectra of the devices were carried out in ambient atmosphere without further encapsulations.

### 2.2. Computational details

The optimized geometry, HOMO and LUMO contour map of Ir(NPIBT)_2_ (acac) were studied with Gaussian-09 package [DFT/B3LYP/6-31G (d,p)].^31^

### 2.3. Synthesis of 5-(1-(naphthalene-1-yl)-1*H*-phenanthro[9,10-*d*]imidazole-2-yl) benzene-1,2,3-triol (NPIBT)

**Scheme 1:**
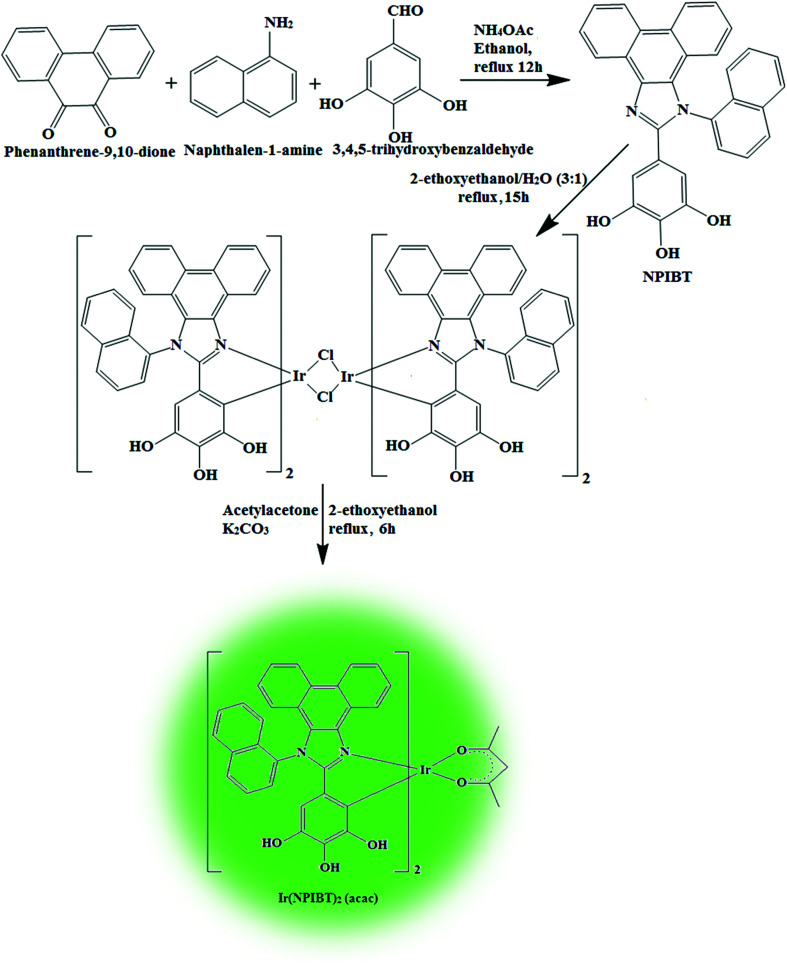
Synthetic route for emissive material Ir(NPIBT)_2_ (acac).

Mixture of 9,10-phenanthrenequinone (5 mmol), 3,4,5-trihydroxybenzaldehyde (5 mmol), 1-naphthylamine (6 mmol) and ammonium acetate (61 mmol) in ethanol was refluxed (12 h: N_2_ stream). From the chilled solution yellow solid was separated and purification was made by column chromatography (Scheme 1) and characterised by NMR spectra, mass spectrometry and elemental analysis (Fig. S1 and S2†). Anal. calcd C_31_H_20_N_2_O_3_: C, 79.47; H, 4.30; N, 5.98. Found: C, 79.39; H, 4.25; N, 5.88. ^1^H NMR (400 MHz, CDCl_3_): *δ* 5.20 (s, 3H), 6.38 (s, 2H), 7.61 (t, 4H), 7.80–7.89 (m, 6H), 8.13 (t, 2H), 8.92 (d, *J* = 8.0 Hz, 3H). ^13^C NMR (100 MHz, CDCl_3_): *δ* 106.81, 122.93, 124.45, 126.30, 126.71, 127.44, 127.85, 127.93, 128.44, 130.52, 131.75, 132.34, 134.61, 136.13, 149.30, 149.50. MS: *m*/*z*. 468.1 [M^+^]. Calcd 467.9.

### 2.4. Synthesis of iridium(iii)-bis-5-(1-(naphthalene-1-yl)-1*H*-phenanthro[9,10-*d*]imidazole-2-yl) benzene-1,2,3-triol (acetylacetonate) [Ir(NPIBT)_2_ (acac)]

The naphthylphenanthrimidazole (NPIBT) (2.2 mmol) and iridium(iii) chloride trihydrate (1 mmol) in 2-ethoxyethanol : H_2_O (3 : 1) was refluxed (N_2_ stream at 120 °C) and the formed dimer (1 mmol) was refluxed (120 °C under N_2_ stream) with acetylacetone (2.2 mmol) and potassium carbonate (2.5 mmol) in 2-ethoxyethanol (5 mL).^32–37^ The green coloured iridium complex was characterized by NMR spectral techniques (Fig. S3 and S4†). Anal. calcd C_67_H_48_IrN_4_O_8_: C, 65.46; H, 3.94; N, 4.56. Found: C, 65.32; H, 3.88; N, 4.43.^1^H NMR (400 MHz, CDCl_3_): *δ* 1.28 (s, 6H), 1.56 (s, 1H), 3.54 (t, 2H), 5.11 (s, 6H), 6.74 (s, 2H), 7.56–7.63 (m, 9H), 7.85 (d, *J* = 8.0 Hz, 8H), 8.14–8.17 (m, 10H), 8.91 (s, 4H). ^13^C NMR (100 MHz, CDCl_3_): *δ* 28.28, 44.01, 46.21, 106.8, 108.10, 122.51, 124.31, 125.90, 126.73, 127.62, 129.31, 130.45, 132.13, 134.76, 136.02, 136.51, 141.65, 146.54, 147.73. MS: *m*/*z*. 1229.31 [M^+^]. Calcd 1229.29.

### 2.5. Sol–gel preparation of nanocrystalline oxide

To the reaction mixture of titanium isopropoxide (0.1 g) in PVP K-30 (0.01 M, 10 mL) zirconium nitrate (0.1 g) and aq. NH_3_ (1 : 1) was added (30 min: pH 7) and the filtered gel was purified with diluted ethanol, dried at 100 °C (12 h) and calcinated (500 °C: 2 h: heating rate 10 °C/min) to form solid.

## 3. Results and discussion

**Fig. 1:**
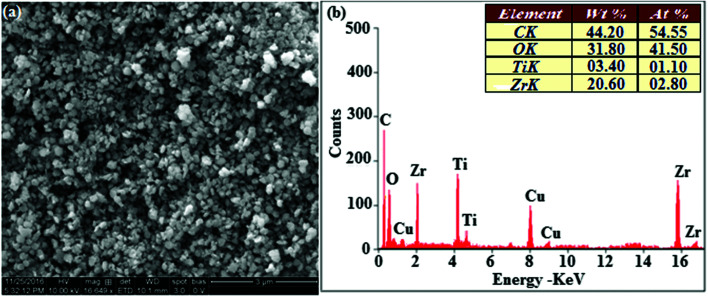
(a) HR-SEM image of Ti-doped ZrO_2_; (b) EDS spectra of Ti-doped ZrO_2_.

**Fig. 2:**
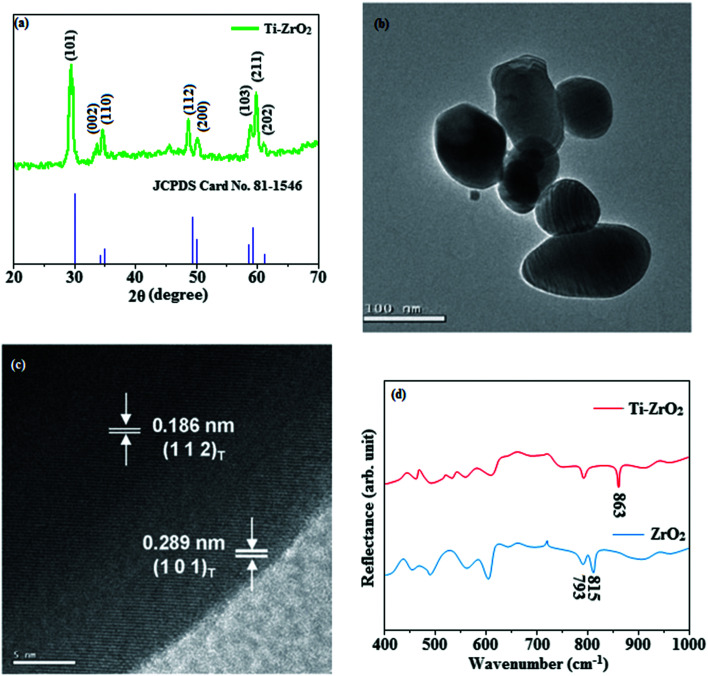
(a) X-ray diffraction pattern of Ti-doped ZrO_2_ (b) & (c) HR-TEM images of Ti-doped ZrO_2_; (d) FT-IR spectra of ZrO_2_ and Ti-ZrO_2_.

**Fig. 3:**
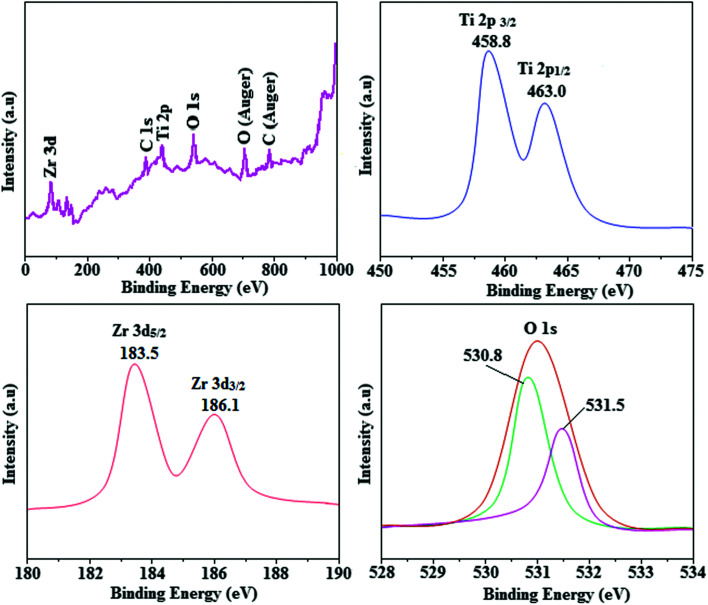
X-ray photoelectron spectra (XPS) of Ti-ZrO_2_.

**Fig. 4:**
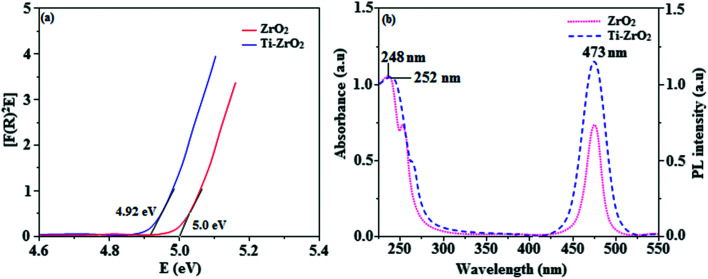
(a) Diffuse reflectance spectra of ZrO_2_ and Ti-ZrO_2_; (b) UV and PL spectra of ZrO_2_ and Ti-ZrO_2_.

The smooth surface of SEM images of Ti-doped ZrO_2_ nanomaterial is due to the use of PVP K-30 templating agent (Fig. 1). The energy dispersive X-ray spectra (EDS) of Ti-doped ZrO_2_ nanomaterials confirm the respective constituent elements and absence of other elements reveal the purity of nanomaterials. Doping percentage of titanium in Ti-doped ZrO_2_ is 28.0 (Fig. 1). The powder X-ray diffraction (XRD) pattern of Ti-doped ZrO_2_ along with JCPDS of tetragonal ZrO_2_ is displayed in Fig. 2 and the observed diffraction pattern matches with that of tetragonal ZrO_2_ (card no. 81-1546). The average crystal size has been deduced using Scherrer equation as 18 nm and the surface area is 58.4 m^2^ g^−1^, respectively. The TEM images of Ti-doped ZrO_2_ reveal the spherical shape nanoparticulate character of Ti-doped ZrO_2_ which is important for display applications. Spherical particles will increase the film brightness and resolution of images because of lower light scattering of emitted light and possess higher packing density compared to irregular shaped particles. The distance between lattice fringes was estimated as 0.289 nm corresponds to 101 plane of tetragonal ZrO_2_ (Fig. 2). Composition of Ti-doped ZrO_2_ was analysed by XPS and the spectrum shows presence of titanium, oxygen and zirconium (Fig. 3). Binding energy peaks of Zr 3d_5/2_ and 3d_3/2_ were observed at 183.5 and 186.1 eV, respectively and are attributed to Zr^4+^.^38^ The doublet observed at 458.8 and 463.0 eV corresponding to Ti 2p_3/2_ and 2p_1/2_ core levels confirm Ti^4+^ in Ti-doped ZrO_2_.^39^ The O1s peak at 530.8 eV is due to bulk oxygen in ZrO_2_ and 531.5 eV may be due to the oxygen of Zr–OH.^40^ DRS spectra of Ti-doped ZrO_2_ and pure ZrO_2_ is compared in Fig. 4: bare ZrO_2_ shows typical absorption at 248 nm due to transition of electron from valence band to conduction band while titanium doped ZrO_2_ show absorption at 252 nm and broad emission at 473 nm which is due to electric transition from conductive band to recombination band (Fig. 4). The broad bandwidth indicates the existence of different recombination sites which was observed in transition metal oxide semiconductors commonly.^41–43^ FT-IR spectra of Ti-doped ZrO_2_ and pure ZrO_2_ show a strong absorption peak at 793 and 815 cm^−1^ corresponds to Zr–O vibrational modes of ZrO_2_ phase (Fig. 2). Moreover, band observed at 863 cm^−1^ corresponds to Ti–O stretching vibration. The observed broad and weaker IR bands are due to the overlapping of Zr–O and Ti–O vibrations of Ti-doped ZrO_2_.^44^ Since, the synthesised Ti-doped ZrO_2_ nanomaterials with band gap (4.92 eV) almost same with ZrO_2_ (5.0 eV: Fig. 4) it could be used as EIL to fabricate HyLEDs to increase of efficiencies.

### 3.1. Characterisation of emissive layer Ir(NPIBT)_2_ (acac)

**Fig. 5:**
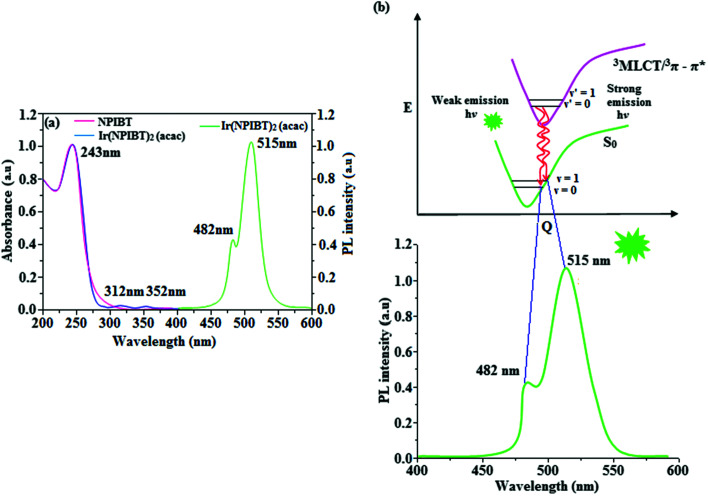
(a) UV and PL spectra of NPIBT and Ir(NPIBT)_2_ (acac); (b) representative Franck–Condon electronic transitions of Ir(NPIBT)_2_ (acac).

**Fig. 6:**
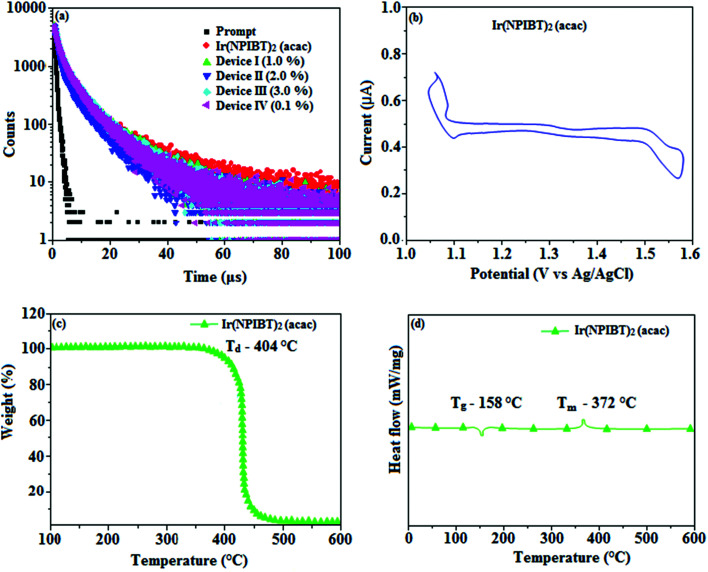
(a) Life time spectra of Ir(NPIBT)_2_ (acac), device I–IV; (b) cyclic voltammogram of Ir(NPIBT)_2_ (acac); (c) TGA and (d) DSC of Ir(NPIBT)_2_ (acac).

**Fig. 7:**
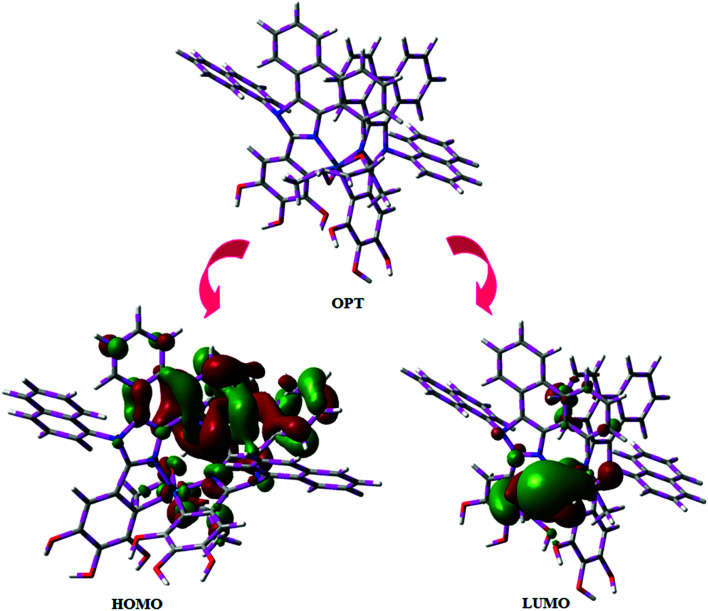
Optimised geometry, HOMO and LUMO contour plot of Ir(NPIBT)_2_ (acac).

Fig. 5 shows UV-Vis absorption (*λ*_abs_) spectra of organometallic complex, Ir(NPIBT)_2_ (acac) in CH_2_Cl_2_ along with free ligand (NPIBT). The intense absorption in the ultraviolet region (243 nm) is at the same energy of NPIBT arises from π–π* transition of the cyclometalated ligand. The other two bands at 312 and 352 nm confirmed MLCT transition (spin allowed) to singlet excited state [^1^MLCT ← S_0_] and triplet excited state [^3^MLCT ← S_0_ ], respectively, both transitions originate from interaction of ligand with iridium center of Ir(NPIBT)_2_ (acac). The intensity of ^3^MLCT ← S_0_ transition is in closest with ^1^MLCT ← S_0_ transition which shows that ^3^MLCT ← S_0_ transition are strongly symmetry allowed by spin–orbit coupling.^45–52^ The spin–orbit coupling was enhanced by closeness of π–π* and MLCT and heavy-atom effect of iridium(iii) of Ir(NPIBT)_2_ (acac). The broad emissive spectra of Ir(NPIBT)_2_ (acac) show green emission at 515 and 482 nm (Fig. 5). The broad spectra reveal the excited triplet states of Ir(NPIBT)_2_ (acac) possess predominantly ^3^MLCT character. Green emitter Ir(NPIBT)_2_ (acac) show strong luminescence both in solution and film from their triplet manifold. Generally, phosphorescence spectra from ligand-centered ^3^π–π* and ^3^MLCT states are in vibronic and broad shape, respectively.^47–50^ Absence of vibronic structured emission spectra of organometallic complex, Ir(NPIBT)_2_ (acac) confirmed the MLCT nature of emission and it is further confirmed by its phosphorescence life time 1.62 μs (Fig. 6). The wave function (*Φ*) of the triplet state (*Φ*_T_) is a mixture of *Φ*_T_ (π–π*) and *Φ*_T_ (MLCT);^53^*Φ*_T_ = *aΦ*_T_ (π–π*) + *bΦ*_T_ (MLCT) [*a* and *b* – normalized co-efficient, *Φ*_T_ (π–π*) and *Φ*_T_ (MLCT) are the wave function of ^3^(π–π*) and ^3^(MLCT) excited states, respectively: when *a* > *b*, triplet state is dominated by ^3^π–π*; when *b* > *a*, triplet state is dominated by ^3^MLCT excited state]. The observed two peaks at 515 and 482 nm attributed to the electronic transition from vibrational level of the triplet state (^3^MLCT/^3^π–π*) to ground state (S_0_) as shown by Franck–Condon electronic transitions (Fig. 5). The peak with dominant intensity stemmed from *ν′* = 0 to *ν* = 0 transition of ^3^MLCT/^3^π–π* to S_0_ whereas a shoulder peak with lower intensity derived from *ν′* = 0 to *ν* = 1 electronic transition.^54–56^ The radiative lifetime of Ir(NPIBT)_2_ (acac) is 1.62 μs and PL quantum yield (*Φ*) is 0.52. The radiative (*k*_r_) and non-radiative (*k*_nr_) decay rate constants have been calculated from the formulae, *Φ* = *Φ*_ISC_ {*k*_r_/(*k*_r_ + *k*_nr_)}, *k*_r_ = *Φ*/*τ*, *k*_nr_ = (1/*τ*) − (*Φ*/*τ*) and *τ* = (*k*_r_ + *k*_nr_)^−1^ [*Φ* – quantum yield; *τ* – lifetime; *Φ*_ISC_ – intersystem-crossing yield].^57–61^ Rate constants reveal that the radiative emission (3.2 × 10^8^ s^−1^) in Ir(NPIBT)_2_ (acac) is slightly predominant over non-radiative transition (3.0 × 10^8^ s^−1^). From DFT [DFT/B3LYP/6-31G (d,p)] analysis, it was shown that the highest occupied molecular orbital (HOMO) is dominantly distributed over d(Ir) and π(C^N) whereas the lowest unoccupied molecular orbital (LUMO) is localized on C^N ligand of the iridium complex (Fig. 7). Ir(NPIBT)_2_ (acac) complex exhibit a distorted octahedral geometry around the iridium atom with two cyclometalated NPIBT ligand and one ancillary acetylacetonate (acac) ligand. The NPIBT ligand adopt eclipsed configuration and two nitrogen atoms N(5) and N(7) reside at *trans*-N,N chelate disposition and the Ir–N distance lie between 2.06 and 2.10 Å. The cyclometalated carbon atoms C(12) and C(21) are mutually *cis* around the iridium atom and Ir–C distance lie between 2.00 and 2.04 Å. Due to stronger Ir–C bonding interaction of the NPIBT ligand which weakens the Ir–C bonds at their *trans* disposition. Electron rich phenyl fragments of Ir(NPIBT)_2_ (acac) shows *trans* effect, thus *trans*-C,C geometry is thermodynamically higher energy and kinetically more labile, called transphobia and it is confirmed by Ir–C bond length Ir–C_av_ = 2.02 Å is shorter than Ir–N bond length, Ir–N_av_ = 2.08 Å.^49,51^ The electrochemical behaviour of Ir(NPIBT)_2_ (acac) exhibit reversible one-electron oxidation wave at *E*_ox_^1/2^ = 0.42 V *vs.* Fc/Fc^+^, which supports the electrochemical stability of the complex (Fig. 6). HOMO energy (−5.20 eV) can be determined from oxidation potential and ferrocenium/ferrocene redox couple energy [*E*_HOMO_ (eV) = −(*E*_ox_ + 4.8)] whereas the LUMO energy (−2.53 eV) is calculated by subtracting the optical band gap energy from HOMO energy [*E*_LUMO_ = *E*_HOMO_ – 1239/*λ*_onset_].^62^ The thermal characterization (*T*_d5_ and *T*_g_) of Ir(NPIBT)_2_ (acac) have been analyzed by DSC and TGA measurements to test its suitability for film formation. The TGA of Ir(NPIBT)_2_ (acac) exhibits high decomposition temperature (*T*_d5_) of 404 °C, high glass transition temperature (*T*_g_) of 158 °C and the melting point (*T*_m_) is 372 °C (Fig. 6). The green emissive material Ir(NPIBT)_2_ (acac) exhibits excellent thermal property and could be subjected to vacuum-evaporation without decomposition.^63–65^

### 3.2. Electroluminescent performances

**Fig. 8:**
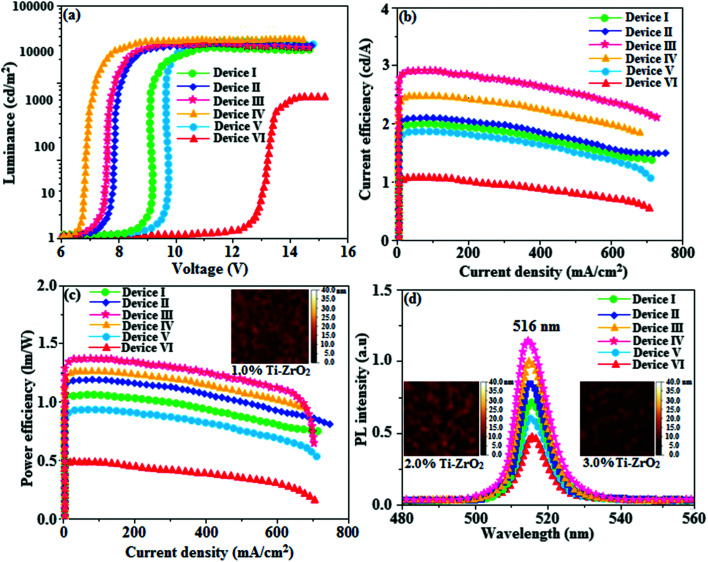
(a) Luminance–voltage of devices I–IV; (b) current efficiency–current density of devices I–IV (c) power efficiency–current density of devices I–IV; (d) electroluminescent spectra of devices I–IV [inset: AFM images of 1.0, 2.0,3.0% Ti-ZrO_2_].

For an efficient HyLEDs device the solid film of the Ti-doped ZrO_2_ nanomaterials should uniformly deposit over the ITO plate and the surface morphology of coated ITO substrates with increasing concentrations of Ti-doped ZrO_2_ (1.0, 2.0 and 3.0%) was analysed through atomic force microscopy (Fig. 8). The thickness and root mean square (RMS) roughness of Ti-doped ZrO_2_ layer from 1.0, 2.0 and 3.0% solutions are 20, 22 and 28 nm and 2.91, 2.74 and 2.42 nm, respectively. The RMS roughness of Ti-doped ZrO_2_ films was much smoother than that of ITO (4.47 nm) and it was slightly decreased as the concentration of Ti-doped ZrO_2_ increased. The absorption edge of Ti-doped ZrO_2_ film was observed at 252 nm (4.92 eV, Fig. 4) which is not band gap of semiconductor and is difference between conduction band edge of Ti-doped ZrO_2_ and LUMO of Ir(NPIBT)_2_ (acac) that determined the injection barrier. The average lifetime (*τ*) (Fig. 6) obtained from decay curves are summarized in Table 1. As thickness of Ti-doped ZrO_2_ layer increases the lifetime also increases [1.41 ns (0%); 1.94 ns (1.0%); 2.01 ns (2.0%) and 2.29 ns (3.0%)]. The 3.0% wt Ti-doped ZrO_2_ blocks exciton quenching efficiently by surface quenching or nonradiative energy transfer quenching mechanisms. The current density–voltage and luminance–voltage variation of the devices with various Ti-doped ZrO_2_ concentrations as well as control device are shown in Fig. 8. The current density and luminance increases significantly by nano Ti-doped ZrO_2_ layer (I–III) compared to control devices IV (0% Ti–ZrO_2_) and V(3% ZrO_2_). The 3.0% Ti-doped ZrO_2_ nanoparticles exhibit maximum luminance of 26 432 cd m^−2^ at driving voltage 7.0 V whereas luminance of 1469 cd m^−2^ at 12.0 V was harvested from control devices IV and V. The device with 3.0% Ti-doped ZrO_2_ layer also shows higher current efficiency *η*_c_ (2.04 cd A^−1^) and power efficiency *η*_p_ (1.15 lm W^−1^) than those of control device (IV). These higher efficiencies reveal that the Ti-doped ZrO_2_ layer in combination with Ir(NPIBT)_2_ (acac) makes electron injection more efficiently through the improved energy level matching at the interface between ITO and Ir(NPIBT)_2_ (acac). The current density and luminance increases as the concentration of Ti-doped ZrO_2_ increases because the total thickness of the device increases. As the thickness of the Ti-ZrO_2_ layer increases up to 4.0%, the current density and luminance slightly increases. This may be due to the fact that the injection and transport of electrons become better as the surface coverage of Ti-ZrO_2_ on top of ITO becomes better with increasing Ti-ZrO_2_ layer thickness and the Ti-ZrO_2_ layer has relatively higher electron mobility than organic materials.^66–68^ The systematic study for the device optimization by controlling the thickness and morphology of Ti-ZrO_2_are under way. Therefore use of Ti-doped ZrO_2_ layer in an optoelectronic device is of current interest owing to advantages of process ability at low temperature, surface roughness and photostability. The band gap energy of TiO_2_ is much lower (3.2 eV) than that of ZrO_2_ (5.0 eV) and this may probably be the reason for effective performances of Ti^4+^ doped zirconia used as electron injection material. The lowering of band gap results in lowering of the CB level of semiconductor nano oxide Ti-ZrO_2_. The reduced potential drive required for promotion of electron from ITO to CB edge of the synthesised nanomaterial reflects the enhanced performances of devices. The Ti-doped ZrO_2_ layer inject electron efficiently due to improved energy level matching at ITO/Ti-doped ZrO_2_/emitting layer interface. Comparing the performances of the HyLEDs with different Ti-doped ZrO_2_ layer thicknesses, a higher efficiency was obtained in the device with thicker Ti-doped ZrO_2_ film because the ability of electron injection becomes improved and the Ti-doped ZrO_2_ layer has relatively higher electron mobility than organic materials. As more electrons injected, the electron–hole balance is enhanced results higher efficiencies than that of control device. Holes are injected from Au anode coated with MoO_3_ HIL into highest occupied molecular orbital (HOMO) of Ir(NPIBT)_2_ (acac) and electrons are injected from Ti-doped ZrO_2_-EIL-coated ITO cathode into LUMO of Ir(NPIBT)_2_ (acac). As conduction band of Ti-doped ZrO_2_ is situated higher than LUMO of emissive material Ir(NPIBT)_2_ this leads to activationless electron injection from metal-oxide into emissive material. The deeper valence band of Ti-doped ZrO_2_ should results efficient hole blocking functionality results improved efficiencies.^30^ The performances of devices along with thickness of Ti-doped ZrO_2_ layer are summarized in Table 1. The normalized EL spectra of HyLEDs (Fig. 8) shows emission around 516 nm measured at the current density of 5.1 mA cm^−2^ and all the EL spectra are in the same shape irrespective of thickness of Ti-doped ZrO_2_ layer since the layer is highly transparent. The use of Ti-doped ZrO_2_ as EIL may due to an lower energy barrier between the conduction level of Ti-doped ZrO_2_ and LUMO of Ir(NPIBT)_2_ (acac) and better hole-blocking ability of Ti-doped ZrO_2_. The efficiencies of newly synthesised electron injection layer of Ti-ZrO_2_ are compared with those of various recently reported electron injection layer (Table S1†).^19,30,69^ It can be seen that the performances of electron injection layer of Ti-ZrO_2_ based devices are among the best in terms of power and current efficiencies and we believe that adopting Ti–ZrO_2_ nanoparticles as EIL is meaningful, a lot in terms of good potential candidate for future displays as well as the device performances.

**Table 1:** Device performances of HyLEDs/Ti-ZrO_2_ (I–IV)/ZrO_2_ (V)/without Ti-ZrO_2_ (VI)/Ir(NPIBT)_2_ (acac)/MoO_3_/Al

% Ti-ZrO_2_	*V*(v)	*L* (cd m^−2^)	*η*_c_ (cd A^−1^)	*η*_p_ (lm W^−1^)	*τ* (ns)
Device I (1.0)	8.6	24 230	1.93	1.03	1.41
Device II (2.0)	7.2	24 948	2.84	1.32	2.01
Device III (3.0)	7.0	26 432	2.04	1.15	2.29
Device IV(4% TiZrO_2_)	6.5	26 996	2.41	1.23	2.41
Device V (ZrO_2_)	9.0	22 401	1.81	0.90	1.23
Device VI (0% TiZrO_2_)	12.0	1469	0.98	0.43	1.94

## 4. Conclusion

In conclusion, the efficient HyLEDs have been fabricated using ITO/Ti-doped ZrO_2_ nanomaterials as a transparent cathode. The device with 3.0% nano Ti-doped ZrO_2_ layer shows higher efficiencies of *η*_c_ (2.04 cd A^−1^) and *η*_p_ (1.15 lm W^−1^) at lower driving voltage compared to control device due to improved energy level alignment. As more electrons are injected to the emitting layer from 3.0% Ti-doped ZrO_2_, electron–hole balance becomes to be improved and hence the higher efficiencies. Ti-doped ZrO_2_ is a good potential candidate for use as EIL in hybrid organic–inorganic light-emitting diodes due to better hole-blocking ability of Ti-doped ZrO_2_.

## Conflicts of interest

There are no conflicts of interest.

## Supplementary Material

RA-008-C8RA00259B-s001
