# Supplementary material for: A hybrid inorganic–organic light-emitting diode using Ti-doped ZrO2 as an electron-injection layer
Source: RSC Adv. 2018 Feb 22;8(15):8402–11. doi: 10.1039/c8ra00259b (PMC9078518; doi:10.1039/c8ra00259b)

Electronic Supplementary Material (ESI) for RSC Advances.
This journal is © The Royal Society of Chemistry 2018

Supporting information

A Hybrid Inorganic–Organic Light-Emitting Diode using Ti-doped ZrO_2 as an Electron-Injection Layer

Jayaraman Jayabharathi*, Sekar Panimozhi, Venugopal Thanikachalam, Annadurai Prabhakaran and Palanivel Jeeva

Department of Chemistry, Annamalai University, Annamalainagar 608 002, Tamilnadu, India.

* Address for correspondence

Dr. J. Jayabharathi
Professor of Chemistry
Department of Chemistry
Annamalai University
Annamalai nagar 608 002
Tamilnadu, India.
Tel: +91 9443940735
E-mail: jtchalam2005@yahoo.co.in

Contents

1. Table S1

2. Figures S1-S4

Table S1: Summary of device performances1-4 and other recently reported green emitting devices

**Electron injection materials**	**L (cd/m²)**	**V (V)**	η_c **(cd A⁻¹)**	η_p **(lm W⁻¹**	**Reference**
ZnO /F8BT	8600	6.0	1.5	-	19
TiO_2 /F8BT	805	10.6	0.03	-	30
PE/Al	1964	0.82	-	-	69
ZrO2/redF	3450	11.8	0.47	0.18	30
ZrO2/L-green	4560	11.8	1.01	0.41	30
ZrO2/L-Blue	470	13.2	0.12	0.03	30
ZrO2/ F8BT	25970	9.0	2.71	1.02	30
ZrO2/PFO	309	9.2	0.03	0.015	30
PE/Ag	1689	0.80	-	-	30
TiO_2 /F8BT	805	10.6	0.03	0.01	30
TiO_2/redF	168	12.4	0.01	0.003	30
TiO_2/L-green	2.9	10.8	0.0002	0.00007	30
TiO_2/L-Blue	5.1	14.8	0.0007	0.0002	30
TiO_2/ F8BT	805	10.6	0.03	0.01	30
TiO_2/PFO	0.1	12.8	0.000005	0.000001	30
Ti-ZrO_2 (1%)	24230	8.6	1.93	1.03	This work
Ti-ZrO_2 (2%)	24948	7.2	2.84	1.32	This work
Ti-ZrO_2 (3%)	26432	7.0	2.04	1.15	This work
Ti-ZrO_2 (4%)	26996	6.5	2.41	1.23	This work

Reference

[19] M. Takada, S. Furuta, T. Kobayashi, T. Nagase, T. Shinagawa, M. Izaki and H. Naito, *J. Appl. Phys.* 2016, **120**, 185501-6.

[30] N. Tokmoldin, N. Griffiths, Donal D. C. Bradley and Saif A. Haque, *Adv. Mater.* 2009, **21**, 3475-3478.

[69] S. Stolz, Y. Zhang, U. Lemmer, G. H. Sosa and H. Aziz, *ACS Appl. Mater. Interfaces.* 2017, **9**, 2776-2785.

Figure S1. ^{1}H **NMR spectrum of 5-(1-(naphthalene-1-yl)-1H-phenanthro[9,10-d]imidazole-2-yl) benzene-1,2,3-triol (NPIBT)**

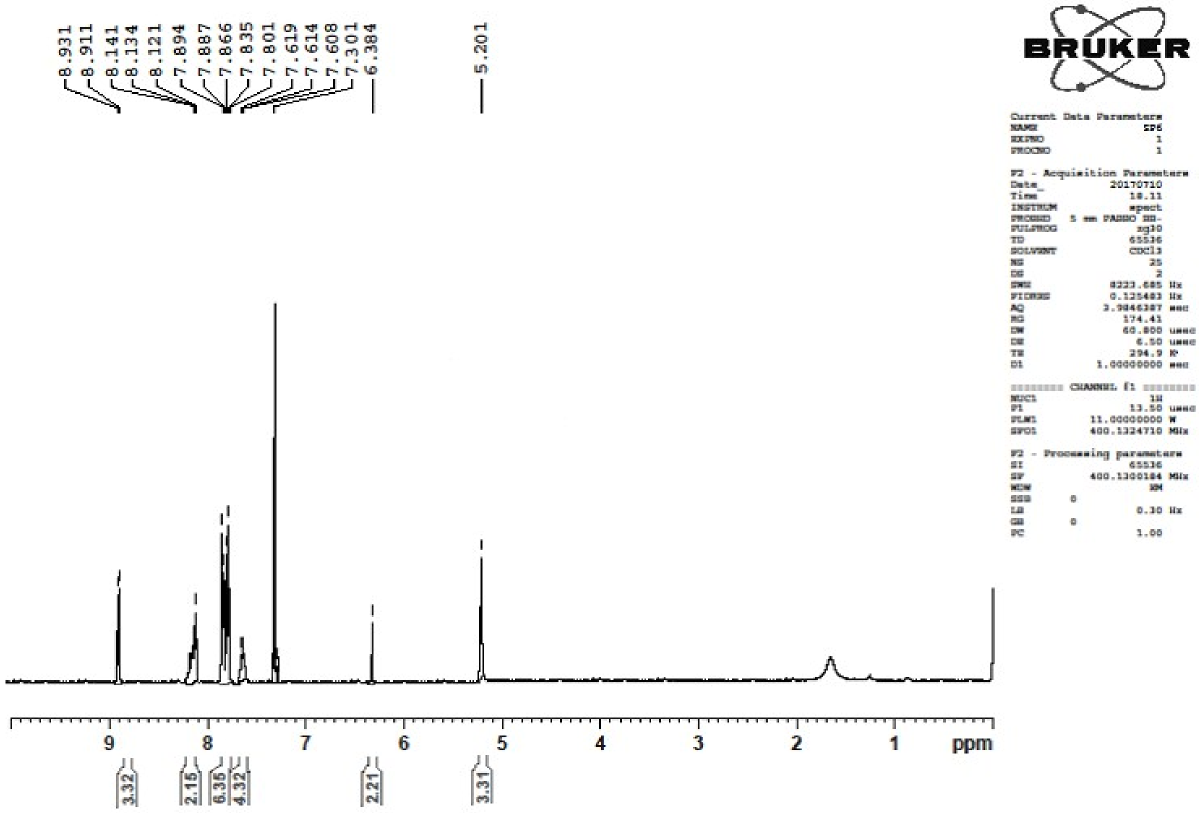

Figure S2. ^{13}C **NMR spectrum of 5-(1-(naphthalene-1-yl)-1H-phenanthro[9,10-d]imidazole-2-yl) benzene-1,2,3-triol (NPIBT)**

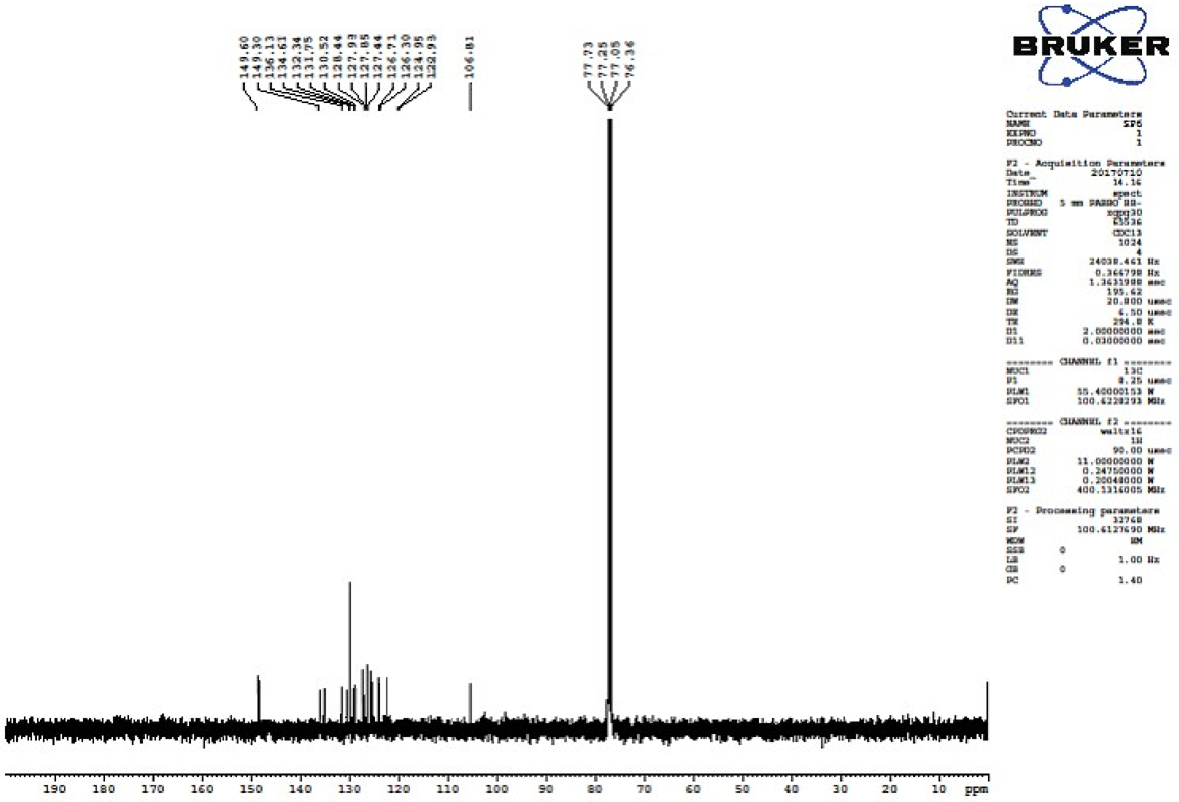

Figure S3. ^{1}H **NMR spectrum of iridium(III)–bis–5-(1-(naphthalene-1-yl)-1H-phenanthro[9,10-d]imidazole-2-yl) benzene-1,2,3-triol (acetylacetonate) [Ir(NPIBT)$_2$ (acac)]**

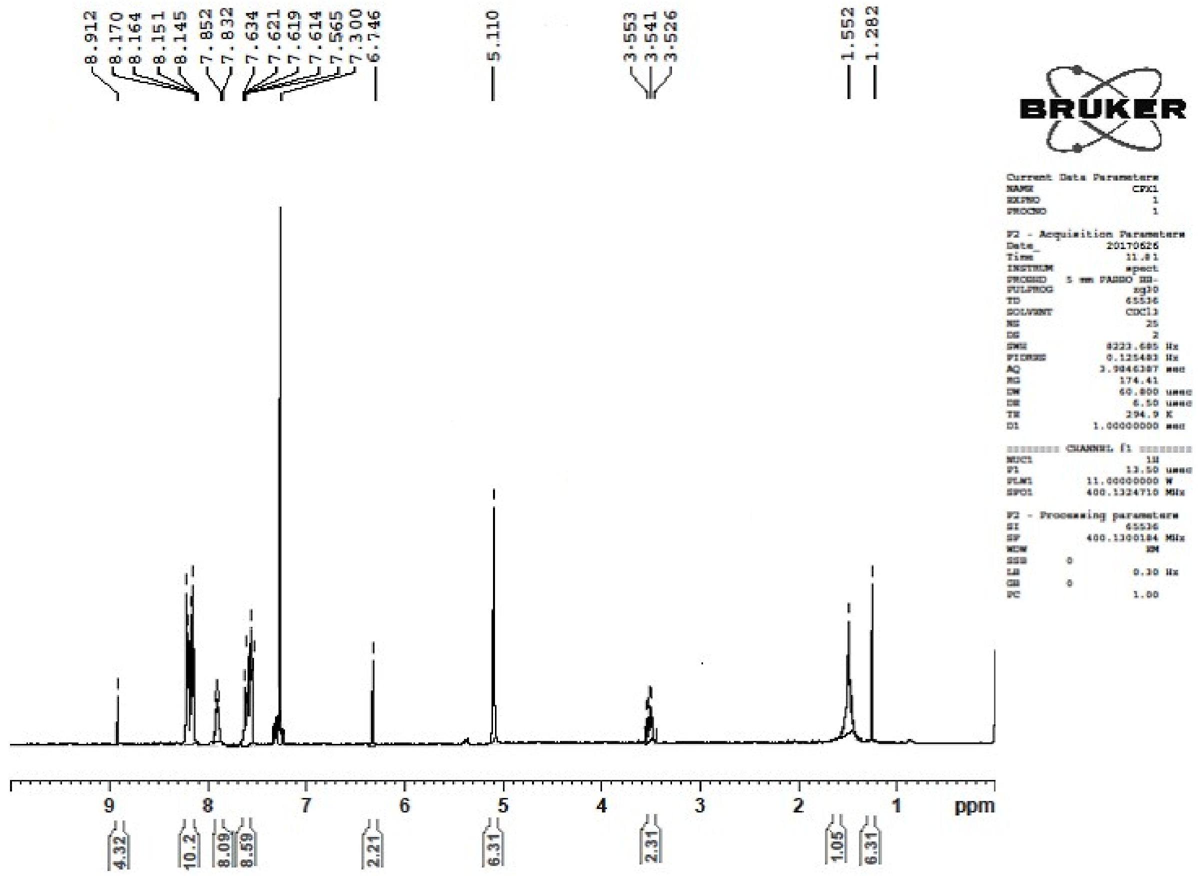

Figure S4. ^{13}C NMR spectrum of iridium(III)–bis–5-(1-(naphthalene-1-yl)-1H-phenanthro[9,10-d]imidazole-2-yl) benzene-1,2,3-triol (acetylacetonate) [Ir(NPIBT)$_2$ (acac)]

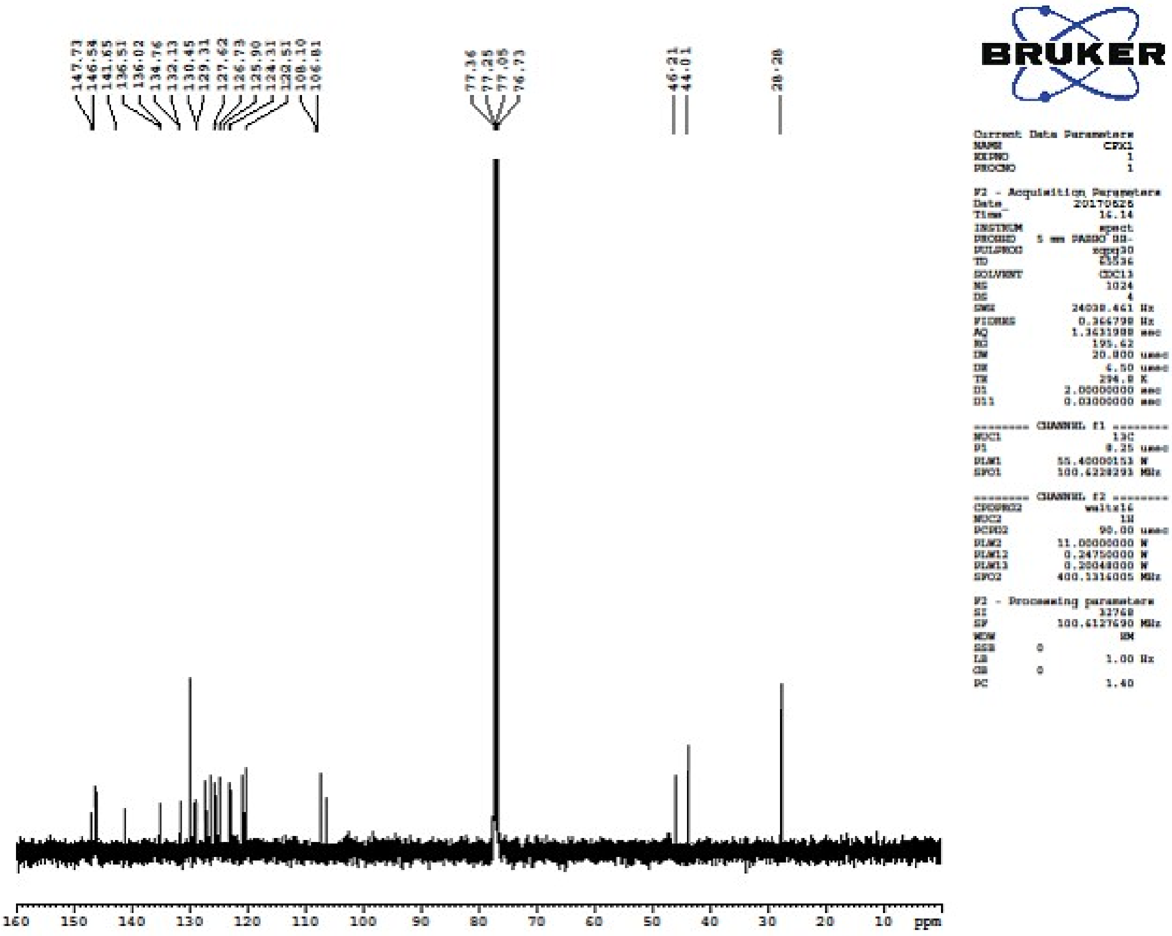

Supplement: RA-008-C8RA00259B-s001 [file RA-008-C8RA00259B-s001.pdf]
